# Anti-Inflammatory Mechanism of Neural Stem Cell Transplantation in Spinal Cord Injury

**DOI:** 10.3390/ijms17091380

**Published:** 2016-08-23

**Authors:** Zhijian Cheng, Wen Zhu, Kai Cao, Fei Wu, Jin Li, Guoyu Wang, Haopen Li, Ming Lu, Yi Ren, Xijing He

**Affiliations:** 1 Department of Orthopedics, The Second Affiliated Hospital of Xi’an Jiaotong University, Xi’an 710004, China; czj.0606@stu.xjtu.edu.cn (Z.C.); caokai@foxmail.com (K.C.); wfhnsx@163.com (F.W.); jin_lee1218@163.com (J.L.); wgy1509@126.com (G.W.); lhp-3993@163.com (H.L.); 2 Intensive Care Unit, The First People’s Hospital of Xianyang City, Xianyang 710021, China; zhuwen841008@163.com; 3 Neurosurgery Department, The Second Affiliated Hospital of Hunan Normal University, Changsha 410003, China; lumingcs163@163.com; 4 Department of Biomedical Sciences, Florida State University, College of Medicine, Tallahassee, FL 32306, USA

**Keywords:** neural stem cells, spinal cord injury, macrophage, inflammatory cytokine

## Abstract

Neural stem cell (NSC) transplantation has been proposed to promote functional recovery after spinal cord injury. However, a detailed understanding of the mechanisms of how NSCs exert their therapeutic plasticity is lacking. We transplanted mouse NSCs into the injured spinal cord seven days after SCI, and the Basso Mouse Scale (BMS) score was performed to assess locomotor function. The anti-inflammatory effects of NSC transplantation was analyzed by immunofluorescence staining of neutrophil and macrophages and the detection of mRNA levels of tumor necrosis factor-α (TNF-α), interleukin-1β (IL-1β), interleukin-6 (IL-6) and interleukin-12 (IL-12). Furthermore, bone marrow-derived macrophages (BMDMs) were co-cultured with NSCs and followed by analyzing the mRNA levels of inducible nitric oxide synthase (iNOS), TNF-α, IL-1β, IL-6 and IL-10 with quantitative real-time PCR. The production of TNF-α and IL-1β by BMDMs was examined using the enzyme-linked immunosorbent assay (ELISA). Transplanted NSCs had significantly increased BMS scores (*p* < 0.05). Histological results showed that the grafted NSCs migrated from the injection site toward the injured area. NSCs transplantation significantly reduced the number of neutrophils and iNOS+/Mac-2+ cells at the epicenter of the injured area (*p* < 0.05). Meanwhile, mRNA levels of TNF-α, IL-1β, IL-6 and IL-12 in the NSCs transplantation group were significantly decreased compared to the control group. Furthermore, NSCs inhibited the iNOS expression of BMDMs and the release of inflammatory factors by macrophages in vitro (*p* < 0.05). These results suggest that NSC transplantation could modulate SCI-induced inflammatory responses and enhance neurological function after SCI via reducing M1 macrophage activation and infiltrating neutrophils. Thus, this study provides a new insight into the mechanisms responsible for the anti-inflammatory effect of NSC transplantation after SCI.

## 1. Introduction

Spinal cord injury (SCI) usually results in loss of functions with limited therapeutic opportunities due to demyelination, axonal damage and loss of neurons [1]. SCI is composed of primary injury and secondary injury. The primary injury involves compression and/or contusion to the spinal cord resulting in tissue destruction and necrosis [2,3,4], which is followed by a cascade of pathophysiological processes hours to days later, resulting in secondary injuries, including tardive apoptosis, demyelination of surrounding neurons, the formation of glial scar and persistent inflammation [5,6]. Among all aspects of secondary injury, the inflammatory response in the injured spinal cord is the major cause and leads to the expansion of the lesion and the worsening of functional deficits. Bone marrow-derived macrophages (BMDMs) and spinal microglia/macrophages are the major inflammatory effector cells. After being activated, they can mediate further tissue damage by producing cytotoxic factors, such as reactive nitrogen species, and pro-inflammatory cytokines, including tumor necrosis factor α (TNF-α), interleukin-1β (IL-1β) and interleukin-6 (IL-6).

Neural stem cells (NSCs) are capable of self-renewal and generating neurons, oligodendrocytes and astrocytes [7,8,9]. Recently, NSC transplantation to treat SCI has become one of the hotspots in the spinal cord injury repair research [10]. NSCs are reported to enhance the recovery of locomotor function in mice after SCI [11,12,13], by means of replacing the damaged neurons and secreting neurotrophic molecules [14]. However, increasing data showed that NSCs can promote motor functional recovery by modulating the host environment. The immunoregulatory and anti-inflammatory effects of NSCs have been widely confirmed in vitro and in vivo, especially in the animal model of experimental autoimmune encephalomyelitis (EAE) [15,16,17]. For example, neural stem/progenitor cells (NS/PCs) can convert encephalitogenic T cells into regulatory T cells in vitro. In addition, NSCs promoted functional recovery through reducing the number of the T helper cell 1 (Th1) and increasing the number of regulatory T cells in vivo [18]. More and more studies are working on unraveling the influence of NS/PCs on microglia. Such as, conditioned medium from both primary mouse neural stem/progenitor stem cells (NS/PCs), and the rat NPCs line can increase the phagocytosis capacity of primary microglia and BV2 microglia cells, a cell line derived from mouse microglia [19]. The subtype of microglia also can be impacted by NS/PCs [20]. However, the anti-inflammatory effects of NSCs against SCI and whether this process is conducted by manipulating of macrophage activation remain unclear.

In the current study, we examined the effects of NSC transplantation on inflammatory response and functional recovery during the sub-acute period of SCI. Specifically, we focused on the influence of grafted NSCs on inflammatory cytokines and macrophages within the epicenter of injured spinal cord. Furthermore, by treating BMDMs with NSCs in vitro, we confirmed the regulation of NSCs on macrophages.

## 2. Result

### 2.1. Isolation and Characterization of NSCs

**Figure 1:**
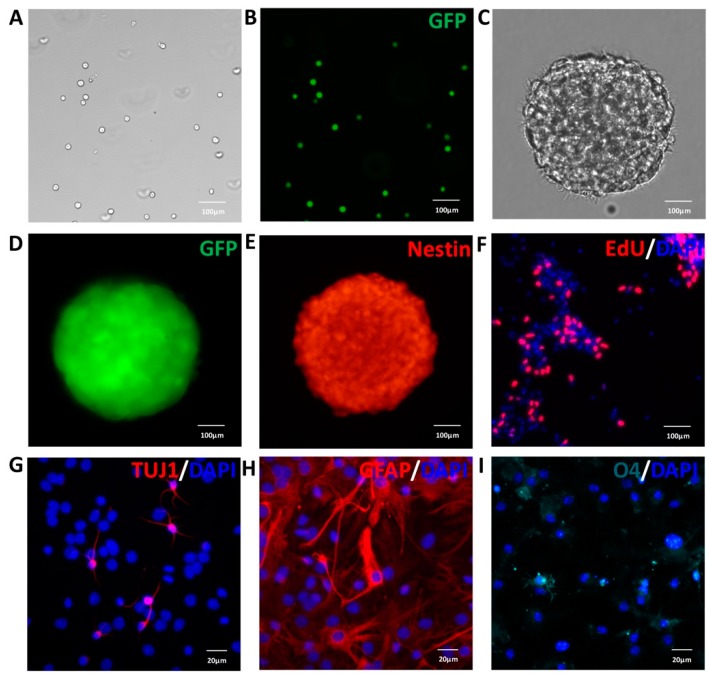
Characterization of neural stem cells (NSCs) from the GFP transgenic mouse in vitro. Mouse NSCs were cultured in growth medium supplemented with 20 ng/mL EGF and 10 ng/mL bFGF. However, to observe the differentiation ability of NSCs, bFGF and EGF were removed, and 1% fetal bovine serum was added into the medium. (**A**) The phase of dissociated NSCs; (**B**) the dissociated NSCs expressed GFP (green); (**C**) the phase of neurosphere; (**D**) neurosphere-expressed GFP (green); (**E**) neurosphere-expressed nestin (red); (**F**) the proliferation of NSCs was determined by cell labeling with 5-ethynyl-2’-deoxyuridine (EdU) (red); (**G**) immunofluorescent staining of the neuronal marker Tuj1 (red); (**H**) the astroglial marker GFAP (red); (**I**) the oligodendrocyte marker O4 (cyan). Nuclei in (**F**–**I**) were stained with DAPI (blue). Scale bar: 100 μm (**A**–**F**); and 20 μm (**G**–**I**).

NSCs from GFP mice embryonic cerebral cortices were isolated and cultured in serum-free medium, including B27, EGF and bFGF. The cells showed strong and stable emission of the green fluorescent signal, proliferated and formed neurospheres (Figure 1A–D). Meanwhile, the neurosphere expressed nestin (Figure 1E), an intermediate filament protein present in neural stem/progenitor cells. To detect the proliferation capacity of NSCs, cells were incubated with 5-ethynyl-2’-deoxyuridine (EdU) for 2 h, and we found that many NSCs were EdU-positive (Figure 1F). Thus, the massive incorporation of EdU showed the efficient proliferation of NSCs. Next, after seven days of differentiation in the medium without EGF and bFGF, the cells expressed the neuronal marker Tuj1 (Figure 1G), the astrocytic marker glial fibrillary acidic protein (GFAP) (Figure 1H) and the oligodendrocyte marker O4 (Figure 1I). Taken together, these results indicated that NSCs used in this study could proliferate, self-renew and exhibit the capacity to differentiate into neurons, astrocytes and oligodendrocytes. The NSCs from passage 3 were used for transplantation and co-culture with macrophages.

### 2.2. Survival, Migration and Distribution of Transplanted NSCs in Host Tissue

**Figure 2:**
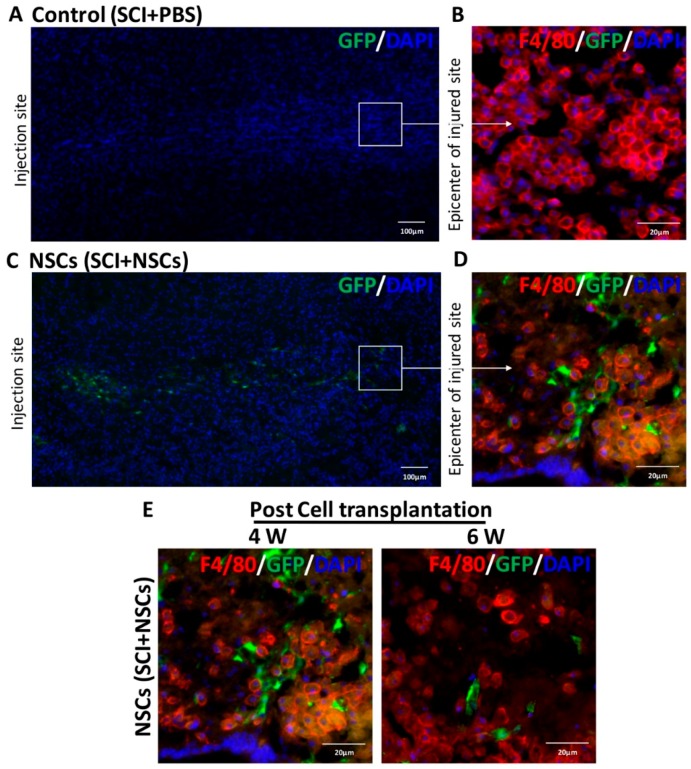
Survival, migration and distribution of grafted NSCs in vivo. (**A**) Mosaic image of a longitudinally-sectioned spinal cord from the control group four weeks post-transplantation; (**B**) only F4/80-positive cells (red) were detected in the epicenter of injury area; (**C**) mosaic image of a longitudinally-sectioned spinal cord from the NSC group four weeks after transplantation; grafted NSCs migrated from the injection site toward injured area; (**D**) in the NSC group, both GFP-positive cells and F4/80-positive cells (red) were found in the epicenter of the injured area; (**E**) representative images of spinal cord sections in the NSC group at four week and six weeks after cell transplantation. Nuclei were stained with DAPI. Scale bar: 100 μm (**A**,**C**); and 20 μm (**B**,**D**,**E**).

Four weeks after transplantation, the migration and distribution of grafted NSCs with green fluorescent protein (GFP) in the injured spinal cord was observed by longitudinal section. Compared to the control group (Figure 2A,B), cells with the GFP signal were found in the area between the implantation site and injured area four weeks after NSC transplantation (Figure 2C). It has been reported that bone marrow-derived macrophages (BMDMs) could migrate to the injured site about three days after the injury and then accumulate at the epicenter of damaged spinal cord [21]. Therefore, the epicenter of the injured area was identified with many F4/80+ cells having a round cell morphology. As shown in Figure 2D, GFP-NSCs arrived at the lesion epicenter, while there were only F4/80-positive cells without GFP-positive cells within the epicenter of the injury area in the control group (Figure 2B). Then, compared to four weeks after NSC transplantation, the number of NSCs within the injured area was significantly decreased at six weeks post-transplantation (Figure 2E). Therefore, these results suggested that grafted NSCs can survive and migrate toward the injured area.

### 2.3. NSCs Transplantation Improved Functional Recovery after SCI

**Figure 3:**
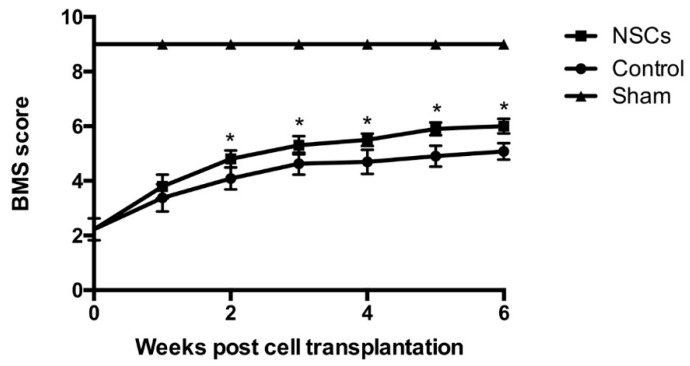
Behavioral assessment and functional recovery by transplanted NSCs after SCI. One week post-SCI, NSCs were transplanted into the injured spinal cord, and Basso Mouse Scale (BMS) scores were recorded weekly. The scores were significantly higher in the NSCs group than in the control group from two weeks to six weeks post-transplantation (*n =* 5 in each time point per group). Data are represented as the mean ± standard error. * *p* < 0.05.

Basso Mouse Scale (BMS) scores were performed at Weeks 1, 2, 3, 4, 5 and 6 post-transplantation to evaluate hindlimb locomotor function. As shown in Figure 3, there was no significant difference of the BMS scores between the control group and the NSC transplantation group at Week 1 post-transplantation (*p* > 0.05; Figure 3). However, BMS scores of the NSC transplantation group were significantly increased compared to the control group from Week 2 to Week 6 post-transplantation (ANVOA, *p* < 0.05; Figure 3). Therefore, our results showed that grafted NSCs can facilitate motor function recovery following SCI.

### 2.4. NSC Transplantation Reduced Neutrophils and Regulated the Activation of Macrophage

**Figure 4:**
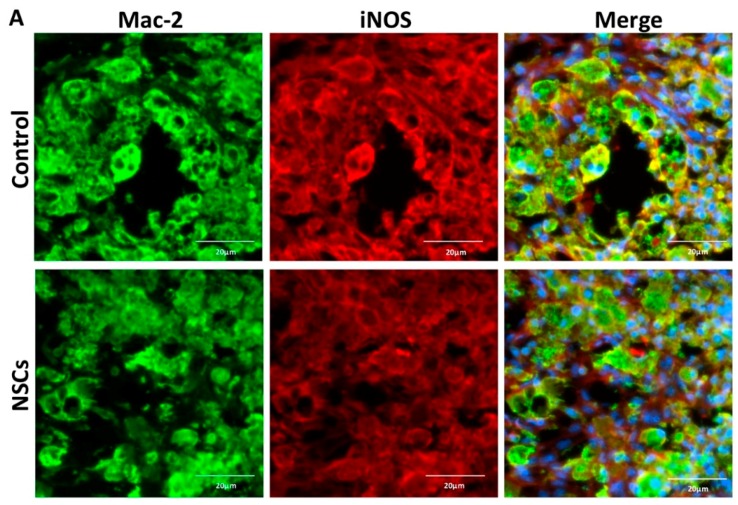

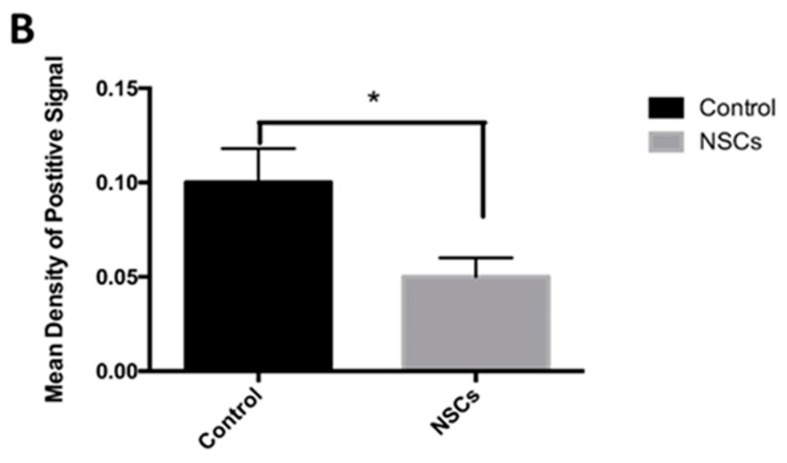
NSC transplantation inhibited macrophages’ activation after spinal cord injury. One week post-transplantation, the injured site was double-stained for iNOS and Mac-2 via immunofluorescent staining. (**A**) Representative images of spinal cord sections immunostained for iNOS (red) and Mac-2 (green) at the injury epicenter; (**B**) quantitative analysis of iNOS and Mac-2 double-positive cells by ImageJ (*n* = 5). Results are displayed as the mean ± standard error. * *p* < 0.05. Scale bar: 20 μm.

To evaluate the effects of NSC treatment on macrophages and neutrophils after SCI, we identified Mac-2-positive microglia/macrophages and anti-neutrophil-positive neutrophils by immunofluorescence staining at seven days after transplantation. As reported, M1 (iNOS-positive) macrophages, pro-inflammatory macrophages, could lead to further damage following primary mechanical injury by secreting inflammatory cytokines, such as TNF-α and chemokines [22]. To determine whether NSC transplantation could promote functional recovery following SCI via regulating the activation of macrophages, we detected the expression of macrophages’ activation marker iNOS. The activated M1 macrophages were identified by double Mac-2 and iNOS staining, and the mean density of the INOS+/Mac-2+ signal was significantly reduced in the NSC group (0.10 ± 0.020) compared to the control group (0.06 ± 0.015) (*p* < 0.05; Figure 4). It suggested that NSC transplantation was able to inhibit the activation of the M1 macrophage, which has been reported to be capable of inhibiting axon extension and to be neurotoxic.

**Figure 5:**
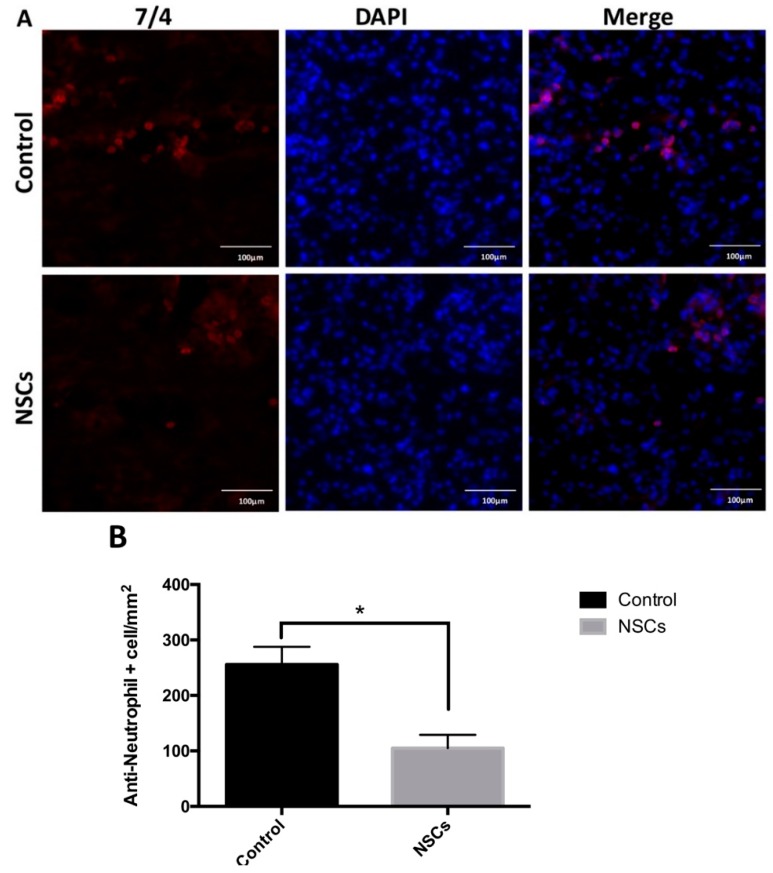
NSCs reduced neutrophil accumulation. (**A**) Representative images of spinal cord sections immunostained for 7/4 (anti-neutrophil antibody) at the injured area one week post-cell transplantation; (**B**) quantification of neutrophil infiltration at the injury site. Positive cells were counted in six sections from each mouse (five mice per group). Data are represented as the mean ± standard error. * *p* < 0.05. Scale bar: 100 μm.

Furthermore, the number of neutrophils (control group: 256 ± 32 cells/mm^2^, NSC transplantation group: 105 ± 10 cells/mm^2^) was significantly decreased one week post-transplantation in the NSC group compared to the control group (*p* < 0.05) (Figure 5).

### 2.5. NSCs Attenuated mRNA Levels of Inflammatory Cytokines in the Spinal Cord Tissue

**Figure 6:**
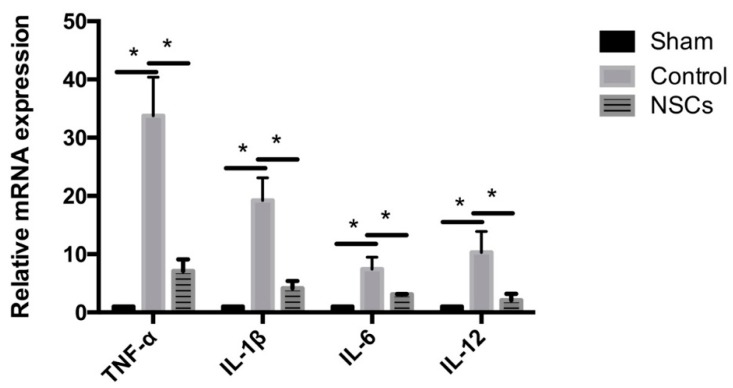
Expression of inflammatory cytokines in spinal cord three days after NSC transplantation. Compared to the sham group, the mRNA levels of TNF-α, IL-1β, IL-6 and IL-12 were significantly increased in the control group. Three days post-transplantation (10 days after SCI), the mRNA levels of TNF-α, IL-1β, IL-6 and IL-12 were significantly decreased in the group transplanted with NSCs, compared to the control group (*n* = 5). Data are represented as the mean ± standard error. * *p* < 0.05.

Next, the expression of tumor necrosis factor-α (TNF-α), interleukin-1β (IL-1β), interleukin-6 (IL-6) and interleukin-10 (IL-10) mRNA in the injured spinal cord tissue of each experimental group was evaluated by using quantitative real-time PCR to determine the effects of NSC transplantation on the expression of inflammatory cytokines in the SCI at three days after transplantation. The results showed that the levels of TNF-α, IL-1β, IL-6 and IL-12 mRNA were significantly higher in the control group than the mRNA levels of the sham group (*p* < 0.05; Figure 6). Compared to the control group, the mRNA levels of TNF-α, IL-1β, IL-6 and IL-12 were significantly decreased in NSC-treated animals (*p* < 0.05; Figure 6).

### 2.6. NSCs Inhibited BMDMs’ Activation and Reduced the Release of Inflammatory Cytokines by Macrophages in Vitro

**Figure 7:**
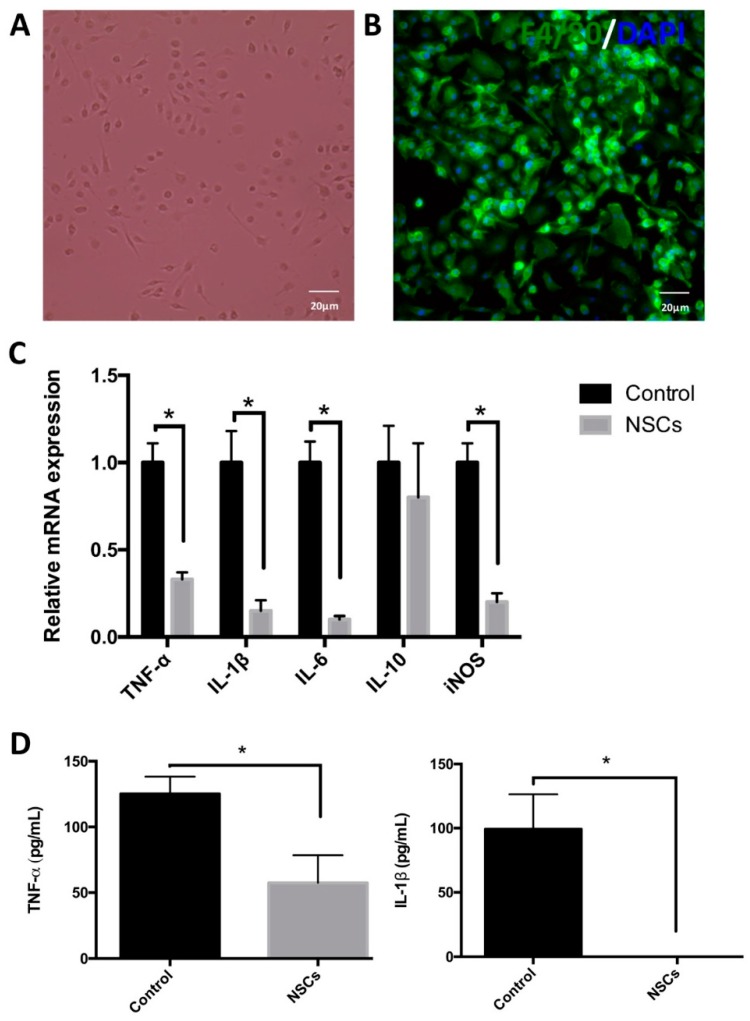
The effects of NSCs on the regulation of macrophage activation in vitro. (**A**,**B**) Mouse bone marrow-derived macrophages (BMDMs) were cultured in DMEM supplemented with 5% newborn calf serum and 15% L929 cell conditioned media for seven days in vitro. (**A**) A phase of the macrophage; (**B**) BMDMs expressed the macrophage marker F4/80 (green), and the nuclei were stained with DAPI (Blue); (**C**) BMDMs were cultured alone or co-cultured with NSCs by using transwells for 12 h, then incubated with 10 ng/mL IFN-γ for 12 h. Next, the mRNA levels of iNOS, TNF-α, IL-1β, IL-6 and IL-10 were detected by quantitative real-time PCR (*n* = 6). Results are displayed as the mean ± SD. * *p* < 0.05; (**D**) BMDMs were treated with or without NSCs for 24 h and then were induced by 10 ng/mL IFN-γ for 24 h. The BMDMs’ supernatants were collected, and the production of TNF-α and IL-1β was examined by ELISA (*n* = 6). Data are represented as the mean ± standard error. * *p* < 0.05. Scale bar = 20 μm.

As reported, macrophages, which accumulated at the injury site, are mainly derived from bone marrow. Therefore, to study the effects of NSCs on macrophages, we isolated and cultured mouse bone marrow-derived macrophages (BMDMs). Cells were cultured in media with granulocyte-macrophage colony-stimulating factor (GM-CSF) for seven days and identified by the expression of F4/80, a ubiquitous microglia/macrophage marker (Figure 7A,B). Next, we co-cultured BMDMs with NSCs for 12 h and then treated with interferon-γ (INF-γ) for 6 h. INF-γ is usually used to induce the activation of M1 macrophages. The mRNA levels of TNF-α, IL-1β, IL-6, IL-10 and inducible nitric oxide synthase (iNOS) were examined by quantitative real-time PCR. We found that NSCs significantly inhibited the expression of the target gene stimulated by interferon-γ (*p* < 0.05; Figure 7C). Finally, we detected the production of pro-inflammatory cytokines, such as tumor necrosis factor α (TNF-α) and IL-1β, by using the enzyme-linked immunosorbent assay (ELISA). The ELISA result revealed that the macrophages co-cultured with NSCs using the transwell system before interferon-γ stimulation, TNF-α and IL-1β secretion induced by interferon-γ were significantly reduced (*p* < 0.05, *n* = 6 wells per group; Figure 7D). Taken together, these results suggested that NSCs have an anti-inflammatory effect via inhibiting macrophage M1 activation and the secretion of inflammatory cytokines in vitro.

## 3. Discussion

In this study, we examined the effects of NSC transplantation on the inflammatory response and functional recovery during the sub-acute period of SCI. Specifically, we focused on the influence of the grafted NSCs on inflammatory cytokines and macrophages within the epicenter of the injured spinal cord. Here, we report that the transplanted NSCs can survive properly and migrate from the injected site toward the injured area effectively. Then, the NSCs suppressed the accumulation of neutrophils and macrophages at the injured area; meanwhile, the activation of macrophages to the M1, pro-inflammatory status was blocked partially, as well as the secretion of inflammatory cytokines by macrophages. Consequently, the functional recovery of SCI was improved efficiently by transplantation of NSCs into the injured spinal cord.

Inflammation is a major contributor to secondary injury and involves increased production of chemokines and cytokines. Pro-inflammatory cytokines, such as TNF-α, IL-1β, IL-6 and IL-12 can be produced by endothelial cells, microglia and astrocytes [23,24,25,26]. Macrophages are another important source of them, especially M1 macrophages. TNF-α is released during the earliest stage of SCI and promotes the inflammatory response by increasing the expression of chemotactic factors, which induce the recruitment of neutrophil and monocytes/macrophages into the injured site [25,27]. Blocking TNF-α by using anti-TNF-α neutralizing antibody at 2 h post-stroke can reduce injured volume and improve neurologic outcomes [28]. IL-1β is another proinflammatory cytokine that could induce the apoptosis of neuronal cells and play an important role in secondary injury. Seventy-two hours after injury treated with the IL-1 receptor antagonist, the contusion-induced apoptosis and caspase-3 activity were significantly reduced [29]. Our study showed that NSC transplantation can create a relatively hospitable homeostasis at the injured area for tissue repair and regeneration by significantly decreasing the mRNA level of TNF-α, IL-1β, IL-6 and IL-12.

Blood monocytes are recruited by primary chemokines and cytokines, such as macrophage chemotactic protein 1 (MCP-1)/chemokine (C-C motif) receptor 2 (CCL2), to the injured area where they differentiate into macrophages 2–3 days post-injury [30,31]. Bone marrow-derived macrophages accumulate within the epicenter of the injured spinal cord and play very important roles in neuro-inflammation. Classical macrophages (M1) and alternative macrophages (M2) are usually seen as the tow primary subsets of macrophages at the injured area. M1 macrophages produce high levels of inducible nitric oxide synthase (iNOS), oxidative metabolites and pro-inflammatory molecules, including TNF-α, IL-1β, IL-6 and IL-12 [32,33]. M1 macrophages could lead to a hostile environment at the injured site and cause damage to healthy cells/tissue. For example, M1 macrophages could block axonal regeneration. When M1 microglia/macrophages were induced by lipopolysaccharide (LPS), this could inhibit neurite outgrowth and induce the growth cone collapse of cortical neurons [34]. In addition, M1 macrophages induce axonal retraction in adult dorsal root ganglion neuron; while alternatively-activated macrophages (M2) are characterized by the expression of arginase-1 (Arg-1) and CD206 and based on their high production of anti-inflammatory cytokines, such as IL-10, low production of pro-inflammatory cytokines, such as IL-1β and IL-12, leading to the suppression of excessive inflammatory response and the repair of damaged tissue [35,36]. As reported, M1 macrophages and M2 macrophages co-exist within injury site at Day 3 after SCI, but only M1 macrophages persist until 28 days post-injury [33]. Our study showed that NSCs were also able to suppress the expression of the pro-inflammatory cytokine TNF-α, IL-1β, IL-6 and M1 macrophages in vivo and to reduce the expression of iNOS, TNF-α, IL-1β and IL-6 by macrophages in vitro. This indicated that NSCs can regulate the activation of macrophages by inhibiting M1 macrophages. Other reports have somewhat confirmed our results. Human primary NSCs attenuated the TNF-α secretion of macrophages under LPS stimulation; however, the production of IL-6 is not inhibited by NSCs [37]. This may be due to the difference of NSCs’ source and stimulation. In our experiment, NSCs were isolated from fetal mouse cortices, and macrophages were induced by IFN-γ after being treated with NSCs, but not LPS. Meanwhile, Kim et al. found that induced neural stem cells (iNSCs) also displayed anti-inflammatory functions, promoting neuroprotection through reducing TNF-α secretion and increasing the release of vascular endothelial growth factor (VEGF), and co-culture with NSCs can reduce the number of apoptotic cells in the cortical neuronal cells induced by macrophages [38].

In vitro experiments with NSCs and bone marrow-derived macrophages (BMDMs) showed that NSCs downregulated the polarization of M1. Data obtained in a transwell system suggested that NSCs may be able to inhibit the polarization of macrophages to M1 through the release of soluble molecules. NSC-derived factors, including tissue inhibitor of metalloproteinase type-1 (TIMP-1), vascular endothelial growth factor (VEGF), transforming growth factor-β (TGF-β), matrix metalloproteinase-9 (MMP-9) and haptoglobin, may play important roles in regulating microglia/macrophages functions and activity [19,39,40]. Recently, exosomes, which are nano-sized extracellular vesicles (EVs) that contain a variety of cargos (e.g., proteins, lipids and nucleic acids), have been shown to have a wide range of biological activities. Ti et al. found that mesenchymal stem cell (MSC)-derived exosomes could upregulate the expression of anti-inflammatory cytokines and the promotion of M2 macrophages activation [41]. In our unpublished data, it was demonstrated that NSC-derived exosomes were able to stimulate morphological change and reduce CD86 (an M1 macrophage marker) expression of BMDMs. However, further research is needed to investigate the specific factors of NSCs that modulate the inflammatory cytokine and macrophage activation.

Managing inflammation after SCI through the manipulation of macrophage function could enhance tissue preservation and promote functional recovery following SCI [42]. Inhibition of the depletion of inflammatory monocyte recruitment, reprogramming macrophages towards the M2 phenotype and blocking the M1 activation pathway have been used to treat SCI [36,43,44]. Blocking M1 macrophages and/or regulating macrophage polarization are helpful for controlling and resolving inflammation after SCI [42]. Meanwhile, increasing studies showed that stem cells, including mesenchymal stem cells (MSCs) [45] and embryonic stem cells (ESCs) [46], have the ability to enhance functional recovery through altering the polarization of macrophages. Nemeth et al. observed that transplantation of human MSCs after SCI promoted functional recovery and modified the inflammatory environment by skewing macrophages into the anti-inflammatory M2 phenotype [47,48]. In addition, our previous data showed that ESC-conditioned media can effectively reduce lipid accumulation, promote an M2-like state and improve functional recovery after spinal cord injury [49]. In the present study, NSC transplantation improved functional recovery following SCI possibly due to the downregulation of M1 macrophages. Therefore, the anti-inflammatory effect of stem cells against SCI via regulating macrophage polarization may be another important mechanism responsible for functional improvement.

## 4. Materials and Methods

### 4.1. Experimental Animals

WT C57BL/6 mice and green fluorescent protein (GFP) transgenic C57BL/6 mice were purchased from Jackson Laboratory (Bar Harbor, ME, USA), and the animals were housed in the pathogen-free Animal Experiment Center of Xi’an Jiaotong University’s college of medicine. To complete this study, ten GFP mice were used for extracting NSCs, and seventy-eight female WT C57BL/6 mice were selected for establishing the SCI model. Seven days after SCI, mice were locally injected either with PBS (*n* = 30) or NSCs (*n* = 30). Three days post transplantation, 5 mice per group were used for quantitative real-time PCR to detect the expression of inflammatory cytokines. Other animals were used for the behavioral study and immunohistochemistry (PBS, *n* = 25; NSCs, *n* = 25). Immunohistochemical evaluation was performed on six spinal longitudinal sections from each mouse (5 mice in each group). All animal experimental procedures were approved by the Xi’an Jiaotong University Ethics Committee. Great efforts were made to minimize animal suffering.

### 4.2. Reagents

All chemicals were purchased from Sigma-Aldrich (St. Louis, MO, USA), and cell culture media were purchased from Invitrogen (Carlsbad, CA, USA), unless otherwise indicated. Basic fibroblast growth factor (bFGF) and epithelial growth factor (EGF) were supplied by Millipore (Billerica, MA, USA). Hybridoma cell lines of F4/80 and Mac-2 were purchased from the American Tissue Culture Collection (ATCC, Manassas, VA, USA). The mouse TNF-α ELISA kit (430901) and mouse IL-1β ELISA kit (432601) were from Biolegend (San Diego, CA, USA). Click-iT^®^ EdU Alexa Fluor^®^ 555 Imaging Kit (C10338) and all secondary antibodies were purchased from Invitrogen (Carlsbad, CA, USA). Primary antibodies used in the experiment are listed in Table 1.

### 4.3. Isolation and Identification of NSCs

Mouse fetal NSCs were generated from the cortex of green fluorescent protein transgenic C57BL/6 mice as described by Reynolds and Weiss [50]. Briefly, embryonic cerebral cortices were collected from Embryonic Day 14 (E14) GFP mice and mechanically dissociated into single cells. The cells were cultured in 50:50 DMEM (Dulbecco’s Modified Eagle’s Medium)/F12 medium, in the presence of 10 ng/mL bFGF, 20 ng/mL EGF and 2% B27 supplement and incubated at 37 °C in a humidified atmosphere with 5% CO_2_. The neurospheres were collected and passaged about every 7 days.

To measure cell proliferation, cells were incubated in 10 mmol/L EdU for 2 h, then fixed with 3.7% formaldehyde in phosphate-buffered saline (PBS) and permeabilized with 0.5% Triton X-100. Cells were then stained with the Click-iT reaction cocktail according to the standard protocol Click-iT EdU Alexa Fluor 555 imaging kit (Life Technologies, Carlsbad, CA, USA), followed by DAPI.

To assess the differentiation potential of NSCs, neurospheres were dissociated into single cells and seeded onto a poly-l-lysine-coated coverslip, then EGF and bFGF were removed from the medium, and 1% fetal bovine serum was added. Cells differentiated for 7 days before being fixed for immunostaining.

### 4.4. Experimental Groups

Seventy-eight specific pathogen-free female adult wild-type C57BL/6 mice, weighing 22.90 ± 2.10 g were used for the animal experiment. Mice were randomly divided into three groups: (1) the sham group (18 mice); (2) the SCI + phosphate-buffered saline (PBS) group (30 mice); (3) the SCI + NSC group (30 mice).

### 4.5. Establishment of the SCI Animal Model

Adult 8–10-week-old female C57BL/6 mice were anaesthetized with ketamine/xylazine, and a laminectomy was performed at the T9-10 vertebral level. Contusion SCI was induced using an NYU impactor with a 5-g rod 6.25 mm onto the spinal cord [51]. Postoperative care consisted of enrofloxacin, buprenorphine and saline once daily for 3 days. Manual bladder emptying was done twice every day, until urinary function was restored. Mice in the sham group were subjected to the same surgical operation process, but no injury.

### 4.6. NSC Transplantation

NSC transplantation was performed similarly to previously described techniques [52]. Briefly, mice with similar BMS scores were randomly assigned to two groups: SCI + phosphate-buffered saline (PBS) (30 mice) and SCI + NSCs group (30 mice). Seven days following SCI, mice were anaesthetized, and the spinal cords were re-exposed. NSCs were resuspended in phosphate-buffered saline (PBS) before transplantation. The viability of NSCs was evaluated by Trypan blue, and cell concentration was adjusted to 1 × 10^5^ cells/1 μL PBS. Each mouse received either GFP NSCs or an equal volume of PBS via microinjecting at 1-mm rostral and caudal to the epicenter. A 0.5-μL suspension was injected at each site. The injections were at a rate of 0.1 μL per minute, and the microsyringe was kept in place for a further 5 min after each injection to minimize the leakage of the cell suspension.

### 4.7. Assessment of Locomotor Function

The locomotor improvement was assessed by three investigators who were blinded to the surgery and treatment group of the tested animals using the Basso Mouse Scale (BMS, 0–9) [53] (sham group, *n* = 13; control group, *n* = 25; NSC group, *n* = 25). A score of 9 indicated unimpaired locomotion as observed in the sham mice. BMS score was conducted from 1 week after SCI until being sacrificed.

### 4.8. Tissue Preparation and Immunohistochemistry

Following transcardial perfusion with 0.9% saline and then fixation with 4% paraformaldehyde in 0.1 mol/L PBS, segments of spinal cord encompassing the injure site were collected and placed in 4% paraformaldehyde for 24 h and then cryoprotected in 30% sucrose overnight at 4 °C. Ten micrometer-thick sections were cut by using a Microm HM505E cryostat (Wayzata, MN, USA). For Immunofluorescence staining, tissue sections from mice (*n* = 5 per group) were blocked in PBS with 1% BSA and 0.3% Triton X-100 for 1 h at room temperature, then incubated with primary antibodies overnight at 4 °C and finally washed and incubated with secondary antibodies at room temperature for 1 h. Non-specific binding was excluded by staining the sample only with secondary antibodies. All images were captured by EVOS FL.

### 4.9. RNA Isolation and Quantitative Real-Time PCR

Messenger RNA (mRNA) levels of TNF-α, IL-1β, IL-6 and IL-12 were measured via quantitative real-time PCR as described elsewhere [54]. Three days after cell transplantation, mice were sacrificed, and spinal cord tissues encompassing the lesion were immediately extracted (*n* = 5 per group). Total RNA was isolated by TRI reagent and reverse transcribed into cDNA by using the fist strand cDNA synthesis kits (Roche, Basel, Switzerland). The following primer sets were as follows: TNF-α (5′-GCCTCTTCTCATTCCTGCTTG-3′ and 5′-CTGATGAGAGGGAGGCCATT-3′), IL-1β (5′-AAGTGATATTCTCCATGAGCTTTGT-3′ and 5′-TTCTTCTTTGGGTATTGCTTGG-3′), iNOS (5′-TTGGAGCGAGTTGTGGATTGT-3′ and 5′-GTAGGTGAGGGCTTGGCTGA-3′), IL-6 (5′-TGGGAAATCGTGGAAATGAG-3′ and 5′-CTCTGAAGGACTCTGGCTTTG-3′), IL-10 (5′-CAACATACTGCTAACCGACTCCT-3′ and 5′-TGAGGGTCTTCAGCTTCTCAC-3′) and GAPDH (5′-ATCAACGACCCCTTCATTGACC-3′ and 5′-CCAGTAGACTCCACGACATACTCAGC-3′). The ABI 7900HT Fast real-time PCR detection system (Applied Biosystems, Grand Island, NY, USA) was used for quantitative inflammatory cytokine mRNA. The gene fold change expression levels were represented by the ratio of target/GAPDH.

### 4.10. Isolation of Macrophage

Bone marrow-derived macrophages (BMDMs) were primary cultured as previously described [55]. BMDMs were harvested from adult 6–8-week-old C57BL/6 mice with 7 days of culture in the cell culture medium, which was Dulbecco’s Modified Eagle Medium (DMEM) (Corning, NY, USA) supplemented with 5% newborn calf serum (NCS) (Rocky Mountain Biologicals, Missoula, MT, USA) and 15% conditional medium from the macrophage-colony stimulating factor secreting L929 fibroblast cell line (ATCC, Manassas, VA, USA). 

### 4.11. Enzyme-Linked Immunosorbent Assay

BMDMs were co-cultured with NSCs for 24 h and then stimulated by IFN-γ for 24 h. The macrophages’ supernatant was collected and analyzed by using the mouse TNF-α and IL-1β ELISA according to the manufacturer’s instructions.

### 4.12. Statistical Analysis

Images were quantified using ImageJ 1.48v (National Institutes of Health, Bethesda, MD, USA), and positive signals were reported as the mean signal density. Statistical data were processed using SPSS software version 22.0 (SPSS, Chicago, IL, USA). Results were reported as the mean ± standard error. The statistical significance between two groups was evaluated by Student’s unpaired *t*-test, and ANOVA was used to assess the multiple group comparisons. The significant level was set at *p* < 0.05.

## 5. Conclusions

In conclusion, we have shown that mouse NSC transplantation significantly enhanced neurological function after SCI. This functional recovery may be due to reduced M1 macrophages’ activation and infiltrating neutrophils. It has been reported that NSC transplantation may promote neural regeneration and functional recovery by means of parasecreting neurotrophic factors and replacing lost neurons. However, our study offers new insight into the mechanisms responsible for the functional improvement following spinal cord injury through the beneficial anti-inflammatory effect of NSCs transplantation; while additional research is needed to investigate the specific factors of NSCs that induce these changes.

## Figures and Tables

**Table 1:** Primary antibodies used in this study.

Antigen	Catalog Number	Dilution	Source
GFAP	Ab53554	1:600	Abcam
iNOS	610329	1:600	BD Biosciences
Nestin	Ab24692	1:300	Abcam
Neutrophil (7/4)	AB53457	1:400	Abcam
O4	MAB1326	1:100	R & D Systems
Tuj1	04-1049	1:400	Millipore
